# An Overview of the Analytical Methods for the Determination of Organic Ultraviolet Filters in Cosmetic Products and Human Samples

**DOI:** 10.3390/molecules26164780

**Published:** 2021-08-06

**Authors:** Izabela Narloch, Grażyna Wejnerowska

**Affiliations:** Department of Food Analysis and Environmental Protection, Faculty of Chemical Technology and Engineering, UTP University of Science and Technology, 3 Seminaryjna Street, 85-326 Bydgoszcz, Poland; izabela.narloch@utp.edu.pl

**Keywords:** analytical methodologies, cosmetics products, human samples, organic ultraviolet filters, sample preparation

## Abstract

UV filters are a group of compounds commonly used in different cosmetic products to absorb UV radiation. They are classified into a variety of chemical groups, such as benzophenones, salicylates, benzotriazoles, cinnamates, p-aminobenzoates, triazines, camphor derivatives, etc. Different tests have shown that some of these chemicals are absorbed through the skin and metabolised or bioaccumulated. These processes can cause negative health effects, including mutagenic and cancerogenic ones. Due to the absence of official monitoring protocols, there is an increased number of analytical methods that enable the determination of those compounds in cosmetic samples to ensure user safety, as well as in biological fluids and tissues samples, to obtain more information regarding their behaviour in the human body. This review aimed to show and discuss the published studies concerning analytical methods for the determination of organic UV filters in cosmetic and biological samples. It focused on sample preparation, analytical techniques, and analytical performance (limit of detection, accuracy, and repeatability).

## 1. Introduction

In recent decades, there has been a progressive increase in UV radiation due to the depletion of the stratospheric ozone layer. This promotes an increase in the number of harmful effects on human health such as skin burns, skin photoaging, damage to the skin’s immunological system, pterygium, or skin cancer [1,2]. Accordingly, the number of personal care products containing UV filters has increased rapidly to protect human skin from damaging exposure to sunlight. The currently estimated volume production of UV filters reaches 26.9 million tons [3]. UV filters are frequently added to all types of personal care products such as lotions, shampoos, creams, aftershave products, make-up products, etc. [4,5,6].

The European Union (EU) Regulation 1223/2009—Cosmetics Regulation defines UV filters as “substances which are exclusively or mainly intended to protect the skin against certain UV radiation by absorbing, reflecting or scattering UV radiation” [7]. UV filters are classified into two groups: organic (chemical) UV filters, which absorb UV light, as well as inorganic (physical) UV filters, which reflect and scatter UV radiation. Chemical UV filters are organic molecules capable of absorbing high UV-A and UV-B range radiation. The UV filters have one or more benzene rings and sometimes are conjugated with carbonyl groups [8]. They can be classified into different groups according to their chemical structure: benzophenone derivatives, p-aminobenzoic acid and its derivatives, salicylates, cinnamates, camphor derivatives, triazine derivatives, benzotriazole derivatives, benzimidazole derivatives, and others (Table 1) [9]. One of the most widely used family of UV filters are benzophenones, in particular BP-3, which in 2012 was classified by the US Environmental Protection Agency (US EPA) as “high production volume chemical” [3]. The scale of the problem of the existence of UV filters in the environment was presented by Astle et al. [3], who performed research among Swiss sunbathers on the use of UV filters during one tourist season. On their basis, it was estimated that about 1249 kg of ethylhexyl methoxycinnamate, 152 kg of octocrylene, 145 kg of 4-MBC, and 122 kg of avobenzene were released into Lake Zürich. Therefore, these compounds are the most frequently determined UV filters.

To protect consumers’ health, the substances that can be used as UV filters in personal care products and their maximum allowed concentrations are strictly defined in each country [8]. The European Union regulations permit the use of 29 UV filters in cosmetics in concentrations ranging from 2 to 25% (Table 1). However, only two are inorganic (titanium dioxide and zinc oxide) [7]. Organic UV filters have a hydrophilic or lipophilic character and most of them are classified as water-resistant [8].

Despite the limitations on their use in UV filters, there are no established official analytical methods for the determination of these compounds in cosmetics products. However, to maintain the safety and adequate effectiveness of products containing UV filters, analytical methods should be developed to control the content of UV filters in them [10].

Moreover, due to the daily use of cosmetics containing UV filters, such compounds are absorbed through the skin into the body, where they can be metabolized and eventually bioaccumulated and/or excreted. The dermal absorption may result in harmful health effects like dermatitis but also more serious effects, such as mutagenic, cancerogenic, and/or estrogenic activity [11]. Therefore, because of the adverse effects of UV filters on human health and their potential bioaccumulation, such biological samples as urine, plasma, breast milk, semen, or tissues must be checked for their presence.

In this context, this review aimed to provide a comprehensive overview of the developments related to the determination of UV filters in cosmetic samples and biological fluids and tissues, with special emphasis on sample preparation and analytical techniques, as well as the achieved detection limits, accuracy, and repeatability.

## 2. Analytical Methods for UV Filter Determination in Cosmetic Samples

### 2.1. Sample Preparation

Cosmetic sample preparation depends on sample type, target analytes, and the technique that is to be used. In general, the preparation of a cosmetic sample does not require a complex pre-treatment sample. This is because the UV filter content in the cosmetic samples is at a sufficiently high level for the sample treatment not to require the extraction and concentration steps. Additionally, in most cases (approximately 90%), liquid chromatography is used for analysis, which enables direct analysis of matrices such as cosmetics. It was alleged that in recent decades the methods of determining UV filters in cosmetics have not been modified too much [11,12].

The initial preparation of the sample consists of dissolving a cosmetic sample in a carefully selected solvent (typically ethanol, methanol, ethyl acetate, water, tetrahydrofuran). The step of dissolving the cosmetic sample may be preceded by homogenisation. Depending on the cosmetic product’s type (i.e., consistency), the next steps in the procedure may include sonicating the sample for a few minutes (5–30 min, 40 °C) [10,13,14,15,16,17,18,19,20,21,22,23,24,25,26,27,28,29,30,31,32,33], magnetic mixing [34,35], mechanical shaking [20,36], vortexing (3–4 min), [25,29,32,37], or centrifuging (1–20 min, 3500–14,800 rpm) [14,19,20,25,27,29,32,33], which can help accelerate the solubilisation. The obtained supernatant is often filtered as well (e.g., 0.45 µm nylon membrane filter) [10,13,14,15,16,17,18,21,22,23,24,25,26,37] and/or evaporated [19,25,27,29,33,38].

These procedures are aimed at completely dissolving the sample or leaching the target analytes (e.g., in case of difficult-to-dissolve samples such as wax-balms, lipsticks, or foundations containing insoluble compounds). The achieved high recoveries (Table 2), amounting from 80 to 113%, confirm the effectiveness of these procedures.

Despite the UV filters being the basic components of the samples, no special extraction techniques are needed. However, some authors proposed the use of extraction techniques such as pressurised liquid extraction [35,38], cloud point extraction [14], dispersive liquid–liquid microextraction [27], or hollow fibre liquid-phase microextraction [19].

### 2.2. Analytical Techniques

Since the UV filters are part of the cosmetic products, their determination by direct measurement without a prior separation step is impossible. As such, chromatography methods are typically used. The most common chromatographic technique for determining UV filters is liquid chromatography; this is because UV filters have very high boiling points. In the majority of publications, the reversed-phase liquid chromatography coupled with a UV/Vis spectrometry detector with a single wavelength or with a diode-array is commonly used for this purpose. The application of a diode-array detector makes it possible to receive the whole UV spectrum for all peaks. The most used stationary phase is the traditional octadecylsilica type (C18), but octysilica (C8) and amide (C16) have been used as well [9]. In the case of reversed-phase separations, the most used solvents include water, methanol, tetrahydrofuran, acetonitrile, or their mixtures. The more environmentally friendly analytical methods include using the ethanol–water mixture in the mobile phase [6,12,19,22]. Isocratic or gradient elution modes are practised as well. Some substances can be added to the eluent to cut back peak tailing, such as acetic acid in the case of BP-3 [14,35]. Such reagents as phosphate, sodium acetate, and ammonium acetate are used for buffering. Hydroxypropyl-β-cyclodextrin is used as a mobile phase modifier to improve the resolution between varied analytes [6].

Therefore, gas chromatography is used in derivatization procedures with silylating reagents that can increase UV filter volatility, as well as sensitivity. Some publications [32,35] describe the use of gas chromatography coupled with mass spectrometry with electron impact, with N,O-Bis(trimethylsilyl) trifluoroacetamide and acetic anhydrite used as the derivatizing reagents.

Apart from liquid and gas chromatography, there are also a few other separation techniques. One of them is micellar electrokinetic chromatography [14,24,25,27], which utilises uncoated silica capillaries and sodium dodecyl sulphate as a surfactant. Others include thin-layer chromatography [22,36,37,41], supercritical fluid chromatography [30,42], and square wave voltammetry [20]. Table 2 shows the published reports on the determination of individual UV filters, including the sample preparation step and the analytical methodology, as well as the results obtained in terms of the limits of quantification, recovery method, and its precision.

## 3. Analytical Methods for UV Filter Determination in Biological Samples

**Figure 1:**
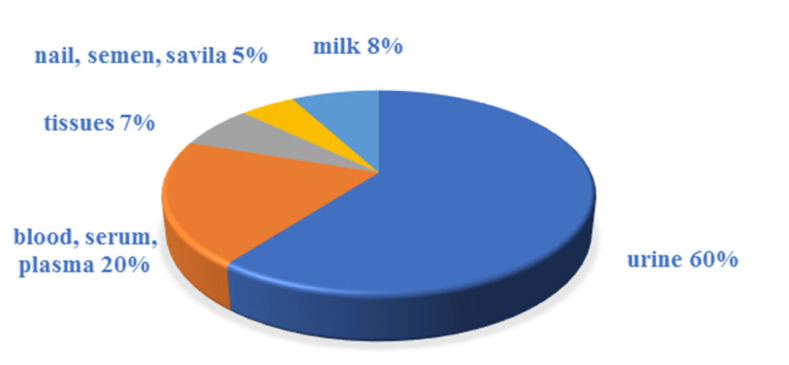
Biological sample types in the determination of UV filters.

Upon classifying published studies dealing with the determination of UV filters in human samples according to the studied matrix (Table 3, Table 4 and Table 5), it is clearly visible that the most studied biological matrix is urine (~61%), followed by blood, plasma, or serum (~20%). Other matrices such as milk (~7%), tissues (~5%), and nail, semen, or saliva (~8%) have only been analysed intermittently (Figure 1).

To date, most research work is focused on the analysis of BP-3 and its metabolites, which have been widely determined in all types of biological samples. Other UV filters that have been analysed, albeit less often, include EMC, OMC, PABA, BDM, EDP, ES, HS, TDS, etc.

### 3.1. Sample Preparation

**Figure 2:**
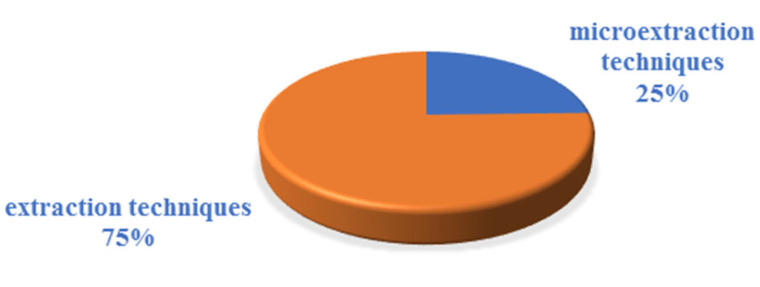
Division of analytical techniques into extraction and microextraction techniques.

To determine UV filters in biological samples, the extraction (~75%) and microextraction (~25%) techniques have been used (Figure 2). Extraction techniques include liquid–liquid extraction (LLE) (~28%), solid-phase extraction (SPE) (~28%), fabric phase sorptive extraction (FPSE) (~5%), as well as the less frequently used accelerated solvent extraction (ASE); microwave-assisted digestion/extraction (MAE); microporous membrane liquid-liquid extraction (MMLLE); matrix solid-phase dispersion (MSPD); sequential injection solid-phase extraction (SI SPE); Quick, Easy, Cheap, Effective, Rugged, and Safe Extraction (QuEChERSExtraction); solid–liquid extraction (SLE); ultrasound-assisted extraction (UAE); and ultrasound-assisted dispersive solid-phase extraction (USAD-SPE) (each ~2%).

**Figure 3:**
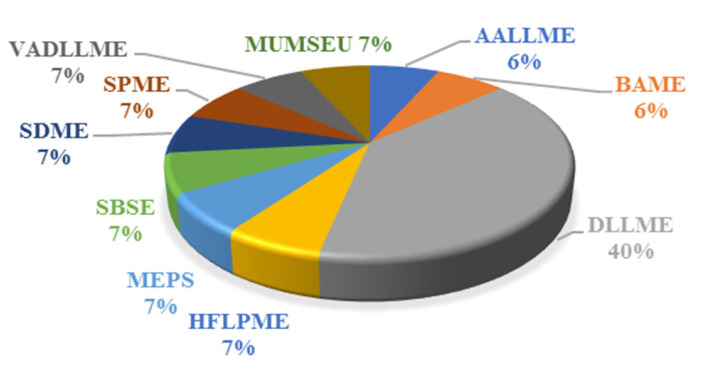
Microextraction techniques used for the determination of UV filters in biological samples.

In the last decades, a gradual increase in the use of microextraction methods for the isolation and enrichment of analytes in the tested samples has been observed. In the work of Jiménez-Díaz et al. from 2014 [43] on methods for determining UV filters in human samples, the contribution of microextraction methods was only about 7%. Microextraction techniques include the dispersive liquid–liquid microextraction (DLLME) (~10%), as well as the less frequently employed air-assisted liquid–liquid microextraction (AALLME), bar adsorptive microextraction (BAµE), hollow-fibre liquid-phase microextraction (HFLPME), microextraction by packed sorbent (MEPS), stir bar sorptive extraction (SBSE), single-drop microextraction (SDME), solid-phase microextraction (SPME), microextraction using a monolithic stirring extraction unit (MUMSEU), and vortex-assisted dispersive liquid–liquid microextraction (VADLLME) (each of them accounts for ~2%) (Figure 3).

Urine is the most frequently analysed sample. In urine, the compounds usually occur in free and conjugated forms; hydrolysis is often required to determine their total content (free plus conjugated). Without the hydrolysis step, it is only possible to determine the content of the free ones. The difference between free and conjugated content gives the total conjugated content. Older studies typically used 6 M hydrochloric acid to hydrolyse the bounded compounds [44,45]. Today, enzymatic hydrolysis is achieved by incubating a urine sample with β-glucuronidase or with β- glucuronidase/sulfatase (under specific conditions such as pH, temperature, and time) [46,47,48,49,50,51,52,53,54,55,56,57,58,59,60,61,62,63,64,65,66,67,68]. After enzymatic hydrolysis, the enzyme is denatured by treated with cold acetonitrile, methanol, or acetic acid to stop the reaction and then separated by centrifugation. The supernatant undergoes the next sample preparation step.

Table 3 summarises the extraction techniques used in the methods for determining UV filters in urine published in the literature. Liquid–liquid extraction (LLE) [51,55,57,58,63,66,69] and solid-phase extraction (SPE) [46,47,48,49,50,56,59,62,64,65,70,71,72] are the most popular extraction techniques used to determine the UV filters. Accelerated solvent extraction (ASE) [62], fabric phase sorptive extraction (FPSE) [73], microporous membrane liquid–liquid extraction (MMLLE) [74], and sequential injection solid-phase extraction (SI SPE) [75] have been employed as well. However, microextraction techniques are also used to reduce solvent consumption and increase concentration factors. Microextraction techniques include air-assisted liquid–liquid microextraction (AALLME) [68], bar adsorptive microextraction (BAµE) [76], dispersive liquid–liquid microextraction (DLLME) [61,77], hollow-fibre liquid-phase microextraction (HFLPME) [55], microextraction by packed sorbent (MEPS) [78], stir bar sorptive extraction (SBSE) [53], single-drop microextraction (SDME) [52], solid-phase microextraction (SPME) [79], and vortex-assisted dispersive liquid-liquid microextraction (VADLLME) [67].

The liquid–liquid extraction is a time-consuming technique, which requires large volumes of organic solvents, and is not automated. It uses different types of organic solvents such as ethyl acetate, a mixture of methyl tert-butyl ether: ethyl acetate, ethanol, methanol, and acetonitrile. The solid-phase extraction is used in manual mode or an online configuration or in commercially available automated workstations. Octadecyl silica sorbents (C18) are widely used for UV filter analysis using SPE in manual mode; divinylbenzene/N-vinylpyrrolidone copolymer (HLB) is an alternative option in this regard. The microextraction techniques are based on the equilibrium processes. Additionally, solid-phase microextraction (SPME) is based on the division of the analyte between the urine sample and a sorbent such as carbowax-DVB fibre. Stir-bar sorptive extraction (SBSE) uses the polymer coating of polydimethylsiloxane as a sorbent. Another microextraction technique is the microextraction by packed sorbent (MEPS), which uses the C18 sorbent to extract analytes. Yet another technique is the dispersive liquid–liquid microextraction (DLLME), which uses solvents (dispersing—acetone and extracting—trichloromethane). Different microextraction methods include hollow-fibre liquid-phase microextraction (HFLPME), based on the use of polypropylene porous hollow fibre, air-assisted liquid-liquid microextraction (AALLME), bar adsorptive microextraction (BAµE), single-drop microextraction (SDME), and vortex-assisted dispersive liquid–liquid microextraction (VADLLME). The final steps are attaining lyophilisation and redissolution of the residue in the solvent.

When examining plasma or serum, blood must undergo additional treatment to isolate them (Table 4). Plasma also includes large proteins such as albumin or immunoglobulin. Such treatment consists in the centrifugation of fresh blood with the addition of an anticoagulant. Serum, however, is prepared by centrifuging blood samples without anticoagulant. To determinate the total compound content, the hydrolysis step must be performed with either acid [81] or an enzyme solution [82,83,84,85]. In the case of blood, serum, or plasma samples, protein precipitation is commonly used to reduce matrix interferences. This is performed by mixing the sample with such organic solvents as acetonitrile [60,63,86], methanol [73,81], acetone [83], or formic acid [84,85]. Proteins are denatured, precipitated, and separated through centrifugation.

The most popular extraction technique in the case of plasma, serum, or blood samples is liquid–liquid extraction with the use of such organic solvents as acetonitrile [60,63,86], as well as a methyl tert-butyl ether [81,87] (Table 4). Another technique is dispersive liquid–liquid microextraction (DLLME) with the use of acetone as the disperser solvent and trichloromethane as the extraction solvent [82,83] or acetone as the disperser solvent and chloroform as the extraction solvent [45]. Solid-phase extraction with the C18 sorbent [84,85] and fabric phase sorptive extraction (FPSE) [73] have also been employed.

In the case of milk, semen, and silva samples, determination takes place in the same way as for urine and plasma samples, and as such, the first step is the acid or enzymatic hydrolysis [69,88,89,90]. Afterwards, acetonitrile [88,90], formic acid [56], isopropanol [89], or methanol [91] is added to precipitate proteins. Finally, in the case of other biological samples such as placenta, nail, or epidermal membrane tissue, homogenisation takes place as well. The samples are shaken and mixed to enable tissue break up (Table 5).

The extraction techniques used in the determination of UV filters in milk, semen, and tissue samples are the same as in the case of urine, i.e., solid-phase extraction, in manual mode [56] and online configuration [89,91]; the ultrasound-assisted dispersive solid-phase extraction (USAD-SPE) is employed as well [92]. Microwave-assisted digestion/extraction (MAE) [93], matrix solid-phase dispersion (MSPD) [94], solid–liquid extraction (SLE) [95], dispersive liquid–liquid microextraction [96], and ultrasound-assisted extraction (UAE) [97] have also been applied for this purpose (Table 5).

### 3.2. Analytical Techniques

Even if an exhaustive initial sample treatment is performed to eliminate possible interfering compounds from the sample, an adequate analytical separation technique must still be selected to improve analyte determination. Table 3, Table 4 and Table 5 present the most used analytical techniques for the detection and quantification of UV filters in biological samples. Liquid chromatography and gas chromatography coupled with MS or MS/MS is the most frequent choice. The choice of either GC or LC is mainly based on the physicochemical properties of the target compounds. GC is usually employed to determinate volatile analytes, whereas LC is applied to quantify both more polar and less volatile compounds.

Liquid chromatography has been used most widely for the determination of UV filters in biological samples. LC coupled with mass spectrometry detectors in tandem is the preferable option. Various ionisation sources have also been used. The most frequently used ionisation mode has been electrospray ionisation (ESI) [45,46,51,55,59,63,64,65,81,82,83,87,90,92,94,95,96,97]. Moreover, it was found that ESI^+^ has better efficiency than ESI^−^ [56]. It is a soft ionisation technique suitable for polar and mildly non-polar compounds. Nevertheless, since ion suppression or improvement in the complex matrix may occur, atmospheric pressure chemical ionisation (APCI) [47,48,49,75,77] and atmospheric pressure photoionisation (APPI) [84,85] have also been used. In all mentioned cases, the determination was carried by multiple reaction monitoring (MRM) mode of the most intense transition, with another one employed to confirm the presence of UV filters in biological matrices at very low concentration levels. Yet another type of detector coupled to liquid chromatography is based on UV/Vis spectroscopy. It is often used due to the fact that UV filters exhibit a high absorbance in the UV range of the electromagnetic spectrum [44,52,60,70,75,76,77,80,86]. Liquid chromatography coupled with a fluorometric detector has been scarcely used because most UV filters do not exhibit fluorescence properties. LC-FL was only used twice—in determining PBSA [71], as well as PEG-25 and PABA [72] in urine samples.

While gas chromatography has been used less often, in most cases it is coupled with mass spectrometry with electron impact [53,54,62,74,79]. In the case of UV filters, a derivatisation step is required before the GC analysis. UV filters have been typically derivatized by using such silylating reagents as N,O-Bis (trimethylsilyl) trifluoroacetamide with trimethylchlorosilane (BSTFA-TMCS) [62] or N-methyl-N-(trimethylsilyl) trifluoroacetamide (MSTFA) [54].

Lastly, despite comprehensive sample preparation and the use of carefully select analytical techniques, it must be noted that final results may sometimes be affected by the “matrix effect.” This phenomenon may impact quantitative recoveries when using external calibration. As such, it may cause differences in the behaviour of the analytes with the accompanying matrix compounds that one can use to enhance or decrease the signal (e.g., ion suppression in the mass spectrum) or affect the extraction efficiency when the extraction technique is used. This negative effect has been adjusted for by using a matrix-matched calibration (the use of the same matrix without analytes to prepare the standard calibration solutions). In other cases, the standard addition calibration method or an isotopic internal standard was used.

### 3.3. Accuracy and Sensitivity

Table 3, Table 4 and Table 5 show information about achieved results for different analytical methods used for the determination of UV filters in biological samples.

The analytical methods presented in it resulted in recoveries enabling exhaustive quantification of the target UV filters in the biological matrices, using external or matrix-matched or standard addition calibration. Thus, in the case of urine samples, the greatest recoveries have been achieved for BP-2 (118%) using microextraction by packed sorbent [78] and for EHS (113%) using liquid–liquid extraction [63]. In the case of blood, plasma, and serum samples, the best recoveries have been obtained for BP-1 (146.4%) using liquid–liquid extraction [81]. In milk samples, the highest-level recoveries have been achieved for BP-3 (112%) by using salt-assisted liquid–liquid extraction coupled with dispersive solid-phase extraction [88]. The recoveries in the case of the determination of OMC in placenta tissue by using ultrasound-assisted extraction amounted up to 112% [97].

In terms of sensitivity, the published methods (Table 3, Table 4 and Table 5) enable the determination of UV filters in the low pg mL^−1^ range.

In the urine samples, the lowest limit of detection (LOD) has been achieved for BP-3 (5 pg mL^−1^) using hollow-fibre liquid-phase microextraction [42]. The LOD for BP-3, 4-MBC, OC, and HS (0.47–0.59 pg mL^−1^) was obtained by using accelerated solvent extraction coupled with solid-phase extraction [62]. In the plasma sample, the LOD was at a level of 0.8 pg mL^−1^ for BP; it was determined using liquid–liquid extraction in conjunction with solid-phase extraction [87].

In the milk sample, the best LOD has been achieved for BP-6 and BP-1 (0.1 ng mL^−1^) using salt-assisted liquid–liquid extraction coupled with dispersive solid-phase extraction [69]. In the determination of 4-OH-BP in the tissue sample, the LOD of 0.02–10 ng mL^−1^ has been obtained using solid–liquid extraction [95].

The low levels achieved in the determination of UV filters in biological samples have been influenced by the use of sensitive analytical techniques (e.g., MS/MS), as well as such enrichment techniques as LLE, SPE, MALLE, SPME, SBSE, SDME, HF-LPME, and MALLME.

## 4. Conclusions

Organic UV filters are a family of cosmetic ingredients most widely used in a common variety of cosmetic products to protect consumers from UV solar radiation. Since compounds belonging to this group can be metabolised, excreted, and/or bioaccumulated, UV filters may be harmful to the human body. This has made analysing UV filters both in cosmetics products and biological samples a necessity.

Liquid chromatography with MS or UV detection is the dominant method for the determination of UV filters. The large majority of published works used conventional C18 or C8 separation columns. Due to the low level of UV filters in the biological samples (e.g., urine, blood, milk), it is necessary to perform the extraction and clean-up steps before the determination procedure to improve the detection limits. LLE and SPE are the most widely used sample preparation and enrichment methods among all those used. However, these conventional techniques present some drawbacks, such as the consumption of large volumes of sample and often toxic organic solvents, but they are time consuming. Nonetheless, such modern microextraction techniques as MEPS, SPME, SBSE, or DLLME are used as well. However, they are only used in 25% of analytical procedures. Due to the trends of modern analytical techniques towards “Green Analytical Chemistry,” they should in the future replace the classic methods of preparing samples for research. This is because of their many advantages, i.e., time-consuming and labour intensity, and above all because they are solvent-free methods.

This review paid special attention to the analytical performance, e.g., limits of detection, accuracy, and repeatability for developed and validated analytical methods. Organic UV filters have been determined to be prevalent in all kinds of biological matrices and are associated with specific markers connected to metabolism, physiological development, and harmful effects in the human body.

## Figures and Tables

**Table 1:** List of compounds that can be allowed as organic UV filters in cosmetic products according to the European Union legislation.

Chemical Name	INCI Name ^a^	Abbreviation	CAS Number	Structure	Max. Concentration (%)	Log K_o/w_^a^	p_Ka_^a^	Solubility (g/L) ^a,b^
**Benzophenone derivatives**								
2-Hydroxy-4-methoxybenophenone/Oxybenzone	Benzophenone-3	BP-3	131-57-7	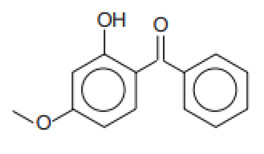	10	3.79	7.56	0.21
2-Hydroxy-4-benzophenone-5-sulfonic acid and its sodium salt/Sulisobenzoate	Benzophenone-4, Benzophenone-5	BP-4, BP-5	4065-45-6/6628-37-1	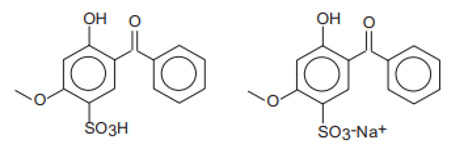	5 (as acid)	0.37	−0.70	0.65
Benzoic acid, 2-[4-(diethylamino)-2-hydroxybenzoyl]-hexylester	Diethylamino Hydroxybenzoyl Hexyl Benzoate	DHHB	302776-68-7	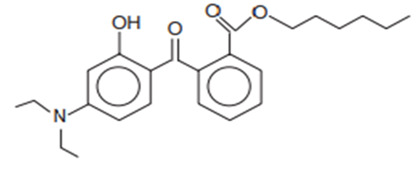	10	6.54	7.29	9.5 · 10^−4^
**p-Aminobenzoic acid derivatives**								
Ethoxylated ethyl-4-aminobenzoate	PEG-25 PABA	PEG-25 PABA	116242-27-4	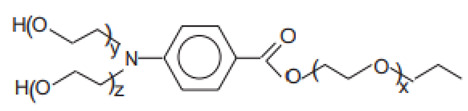	10	−0.66	-	-
2-Ethylhexyl-4-(dimethylamino)benzoate/Padimate O (USAN:BAN)	Ethylhexyl Dimethyl PABA	OD-PABA	21245-02-3	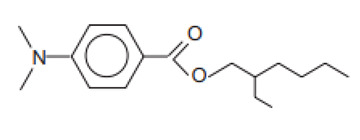	8	6.15	2.39	0.0021
**Salicylates**								
Benzoic acid, 2-hydroxy-3,3,5-trimethylcyclohexyl ester/Homosalate	Homosalate	HS	118-56-9	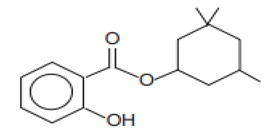	10	6.16	8.09	0.02
2-Ethylhexyl salicylate/Octisalate	Ethylhexyl Salicylate	EHS	118-60-5	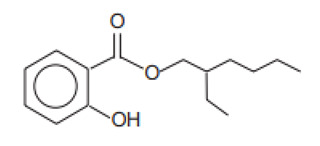	5	5.97	8.13	0.028
**Cinnamates**								
2-Ethylhexyl-4-methoxycinnamate/Octinoxate	Ethylhexyl Methoxycinnamate	OMC	5466-77-3	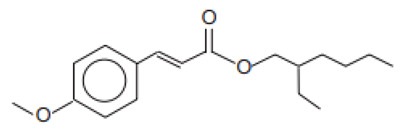	10	5.8	-	0.15
Isopentyl-4-methoxycinnamate/Amiloxate	Isoamyl p-Methoxycinnamate	IMC	71617-10-2	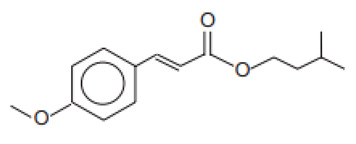	10	4.33	-	0.06
**Benzimidazole derivatives**								
2-Phenylbenzimidazole-5-sulfonic acid and its potassium, sodium, and triethanolamine salts/Ensulizole	Phenylbenzimidazole Sulfonic Acid	PMDSA	27503-81-7	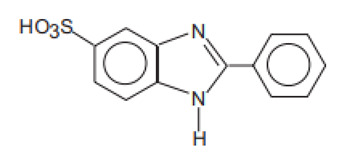	8 (as acid)	−0.16	−0.87	0.26
Sodium salt of 2,2′-bis(1,4-phenylene)-1H-benzimidazole-4,6-disulfonic acid)/Bisdisulizole disodium (USAN)	Disodium Phenyl Dibenzimidazole Tetrasulfonate	DPDT	180898-37-7	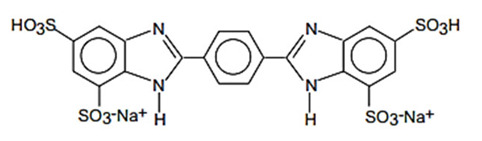	10 (as acid)	−6.79	−0.27	0.5
**Benzotriazole derivatives**								
Phenol,2-(2H-benzotriazol-2-yl)-4-methyl-6-(2-methyl-3-(1,3,3,3-tetramethyl-1-(trimethylsilyl)oxy)-disiloxanyl)propyl)	Drometrizole Trisiloxane	DTS	155633-54-8	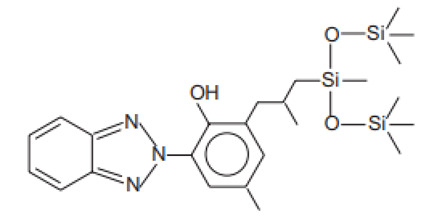	15	10.38	1.2	5.5 · 10^−10^
2,2′-Methylene-bis(6-(2H-benzotriazol-2-yl)-4-(1,1,3,3-tetramethyl-butyl)phenol)/Bisoctrizole	Methylene Bis-Benzotriazolyl Tetramethylbutylphenol	MBP	103597-45-1	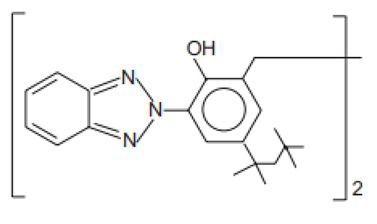	10	12.46	7.56	3 · 10^−8^
**Camphor derivatives**								
N,N,N-Trimethyl-4-(2-oxoborn-3-ylidenemethyl)anilinium methyl sulfate	Camphor Benzalkonium Methosulfate	CBM	52793-97-2	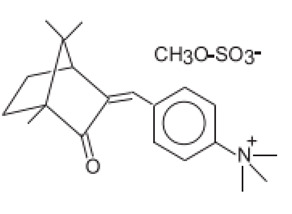	6	0.28	-	0.007
3,3′-(1,4-Phenylenedimethylene) bis(7,7-dimethyl-2-oxobicyclo-[2,2,1]hept-1-yl-methanesu fonic acid) and its salts/Ecamsule	Terephthalylidene Dicamphor Sulfonic Acid	PDSA	92761-26-7, 90457-82-2	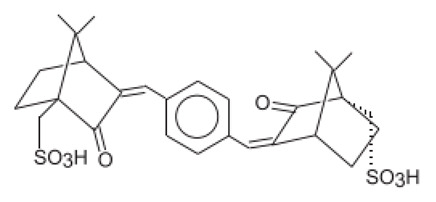	10 (as acid)	3.83	−1.05	0.014
Alpha-(2-Oxoborn-3-ylidene)-toluene-4-sulphonic acid and its salts	Benzylidene Camphor Sulfonic Acid	BCSA	56039-58-8	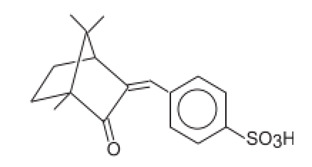	6 (as acid)	2.22	−0.7	0.038
3-(4-Methylbenzylidene)-d1 camphor/Enzacamene	4-Methylbenzylidene Camphor	4-MBC	38102-62-4/ 36861-47-9	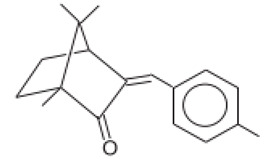	4	4.95	-	0.0051
Polymer of N-{(2 and 4)-[(2-oxoborn-3-ylidene)methyl-]benzyl} acrylamide	Polyacrylamidomethyl Benzylidene Camphor	PBC	113783-61-2	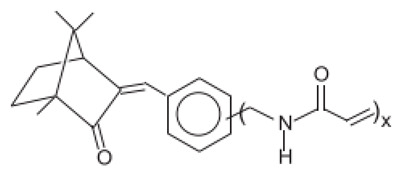	6	-	-	-
**Triazine derivatives**								
Benzoic acid, 4,4-((6-((4-(((1,1-dimethylethyl)amino)carbonyl)phenyl)amino)-1,3,5-triazine-2,4-diyl)diimino)bis-, bis (2-ethylhexyl) ester/ Iscotrizinol (USAN)	Diethylhexyl Butamido Triazone	DBT	154702-15-5	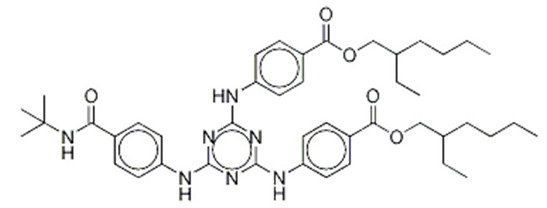	10	14.03	3.04	4.6 · 10^−7^
3,3′-(1,4-Phenylene)bis(5,6-diphenyl-1,2,4-triazine)	Phenylene Bis-Diphenyl triazine	-	55514-22-2	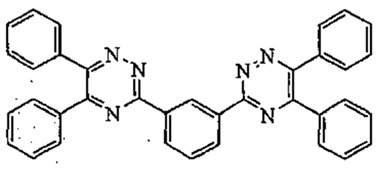	5	-	-	-
2,4,6-Trianilino-(p-carbo-2′-ethylhexyl-1′-oxy)-1,3,5-triazine	Ethylhexyl Triazone	ET	88122-99-0	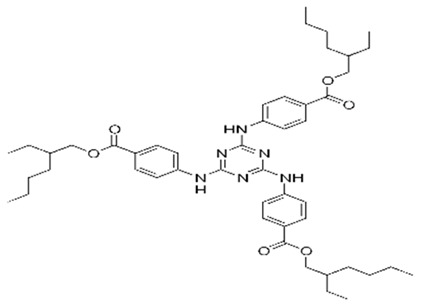	5	17.05	3.17	-
2,2′-(6-(4-Methoxyphenyl)-1,3,5-triazine-2,4-diyl)bis(5-((2-ethylhexyl)oxy)phenol)/Bemotrizinol	Bis-Ethylhexyloxyphenol Methoxyphenyl Triazine	EMT	187393-00-6	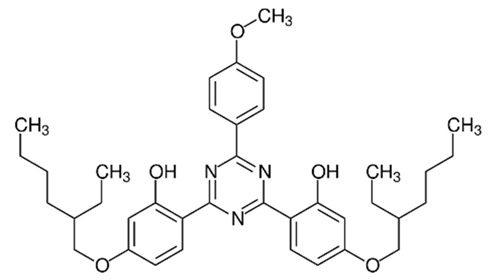	10	8.03	6.37	4.9 ·10^−8^
**Others**								
1-(4-tert-Butylphenyl)-3-(4-methoxyphenyl)propane-1,3-dione/Avobenzene	Butyl Methoxydibenzoyl-methane	BMDBM	70356-09-1	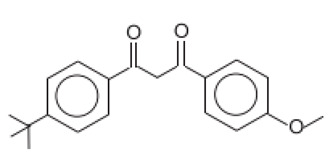	5	4.51	9.74	0.037
2-Cyano-3,3-diphenyl acrylic acid, 2-ethylhexyl ester/Octocrilene	Octocrylene	OC	6197-30-4	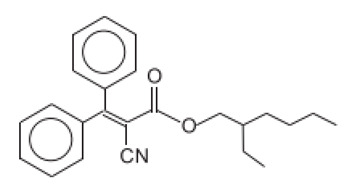	10 (as acid)	6.88	-	2 · 10^−4^
Dimethicodiethylbenzalmalonate	Polysilicone-15	BMP	207574-74-1	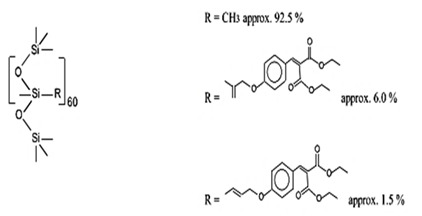	10	-	-	-
2-ethoxyethyl(2Z)-2-cyano-2-[3-(3-methoxy-propylamino) cyclohex-2-en-1-ylidene]acetate	Methoxypropylamino Cyclohexenylidene Ethoxyethylcyanoacetate	-	1419401-88-9	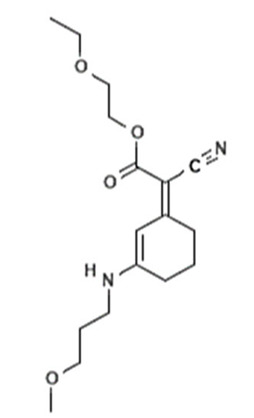	3	-	-	-

^a^ From Cadena-Aizaga M.I. et al. [39]. ^b^ Solubility in water at 25 °C.

**Table 2:** Published studies on UV filters determination in cosmetic samples.

UV Filters	Matrix	Analytical Technique	Analytical Performance ^a^	Ref.
BP-3, IMC, MBC, DHHB, OC, EDP, BDM, EMC, EHS, HS, DBT, ET, DTS, MBP, EMT	Sunscreens, facial creams, lip balms, aftershave creams	LC-UV/Vis; type of column: C_18_; column temperature: 60 °C; mobile phase: ethanol/formic acid (aq) mobile phase modifier: hydroxypropyl-β-cyclodextrin (HP-β-CD)	LOD: 0.02–0.22 µg mL^−1^ LOQ: 0.07–0.74 µg mL^−1^ R: 98–104% RSD: 0.9–7.1%	[10]
PMDSA, BP-4, BP-3, MBC, DHHB, EMC, OC, MBP, EMT, ET, BDM	Emulsion, oil	HPLC-UV/Vis; type of column: C_8_ or C_18_ or C_16_; column temp.: 35 °C; mobile phase: gradient acetonitrile/perchloric acid (aq) or isocratic methanol/acetonitrile or isocratic methanol/perchloric acid	LOD: 0.1–1.2 µg mL^−1^ LOQ: no data R: 93.9–103.4% RSD: 0.2–1%	[13]
BP-1, BP-2, BP-3	Emulsion	MEKC-UV/Vis; type of capillary: a 51 cm uncoated fused-silica; surfactant: sodium tetraborate containing sodium dodecyl sulfate	LOD: 6.42∙10^−8^–3.90·10^−7^ mol/L LOQ: no data R: 89.5–102.5% RSD: 1.14–8.09%	[14]
PMDSA, PABA, BP-4, BP-3, IMC, MBC, OC, EMC, HS, EHS, MBBT	Creams, lotions, foundation, loose powder, lipstick	HPLC-UV/Vis; type of column: C_18_; column temp.: 30 °C; mobile phase: gradient methanol/tetrahydrofuran/perchloric acid (aq)	LOD: 200–500 ng mL^−1^ LOQ: 700–6700 ng mL^−1^ R: 98.5–102.2% RSD: 0.51–1.72%	[15]
PMDSA, BP-3, IMC, DHHB, OC, EMC, EHS, BDM, DBT, ET, MBP, EMT	Emulsion, sticks, powder	HPLC-UV/Vis; type of column: C_18_; column temp.: 40 °C; mobile phase: gradient ethanol/ 1% phosphoric acid (aq)	LOD: 0.04–1.66 µg mL^−1^ LOQ: 0.13–5.52 µg mL^−1^ R: 97–101.4% RSD: 0.38–2.42%	[16]
HS, EDP, EHC, EHS, MBC, BDM, BP-3, OC, PHBA, BC	Cream, milk, lotion, oil, lipstick	DART-MS (ESI^+^)	LOD: 2.5–460 µg g^−1^ LOQ: no data R: 71–120% RSD: 4–30%	[17]
EMC, IMC, EHS, MBC, BP-3, EDP, OC, BDM	Cream, lotion, spray	HPLC-UV/Vis; type of column: C_18_; column temp.: 30 °C; mobile phase: gradient acetonitrile/acetic acid (aq)	LOD: 0.03–1.5 mg L^−1^ LOQ: 0.08–4.6 mg L^−1^ R: 98–102% RSD: 0.97–6.1%	[18]
BP-4, BP-3, ODP, OMC, EHS	Cream, lotion, lipstick, foundation	HPLC-UV/Vis; type of column: C_18_; column temp.: 40 °C; mobile phase: gradient methanol/pure water (80:20; *v*/*v*)	LOD: 1–100 ng L^−1^ LOQ: 4–340 ng L^−1^ R: 98–102% RSD: 4–5.2%	[19]
OC	Emulsion	SWV/mercury electrode; a mixture of Britton–Robinson (BR) buffer and ethanol (7:3; *v*/*v*) as the supporting electrolyte	LOD: no data LOQ: no data R: 9.7–106% RSD: 1–3.42%	[20]
EMC, BP-3, EHS, OC	Emulsion	LC-UV/Vis; type of column: C_18_; mobile phase: methanol/water (85:15; *v*/*v*)	LOD: no data LOQ: no data R: 99.67–101% RSD: 0.044–1.5%	[21]
BDM, BP-3, EMC	Cream	HPTLC-DS.; type of column: C_18 or_ silica gel; mobile phase: acetonitrile/water (18:2) or cyclohexane/diethyl ether/n-hexane/acetone (14:2:1:2)	LOD: no data LOQ: no data R: 92.7–102.4% RSD: no data	[22]
PABA, PMDSA, BP-3, MBC, BP-4, OC, EDP, EMC, BDM, HS, EHS, DBT, ET, DTS	Cream	HPLC-UV/Vis; type of column: C_18_; mobile phase: gradient ethanol/phosphate buffer	LOD: 0.01–1.99 mg L^−1^ LOQ: 0.02–6.02 mg L^−1^ R: 90.91–109.98% RSD: 0.16–12.69%	[23]
BP-3, BP-4	Shampoo, gel, perfume, cream	MEKC-UV/Vis; type of capillary: a 64.5 cm uncoated fused-silica; surfactant: sodium dodecyl sulphate	LOD: 0.91–2.26 µg mL^−1^ LOQ: 2.72–6.79 µg mL^−1^ R: 90.4–107.4% RSD: 5.7–12%	[24]
BP-1, BP-2, BP-3, BP-4, BP-6, BP-8, OC, EMC, PABA	Lotion, cream	MEKC-UV/Vis; type of capillary: a 30.2 cm uncoated fused-silica; surfactant: sodium dodecyl sulfate/γ-cyclodextrin	LOD: no data LOQ: no data R: 95.08–104.57% RSD: no data	[25]
PABA, BP-3, IMC, MBC, OC, EDP, EMC, BDM, EHS, HS	Cream	HPLC-UV/Vis; type of column: C_18_; column temp.: 35 °C; mobile phase: isocratic ethanol/acetic acid (aq) (70:30; *v*/*v*)	LOD: 0.1–2 µg mL^−1^ LOQ: 0.5–5µg mL^−1^ R: no data RSD: no data	[26]
BP, BP-3, BP-1, HBP	Cream	MEKC-UV/Vis; type of capillary: a 60 cm uncoated fused-silica; surfactant: sodium dodecyl sulfate	LOD: 3.9–6.7 ng mL^−1^ LOQ: 13–22.3 ng mL^−1^ R: 80.2–117.7% RSD: no data	[27]
BP-3, EMC, OC, EHS, MBC, EDP	Cream, lipstick, blemish balm cream	LTP-MS	LOD: no data LOQ: no data R: no data RSD: 0.8–28.6%	[28]
PMDSA, BP-2, BP-1, BP-8, BP, BP-6, BP-3, EHS, BP-10, HS, IMC, MBC, DHHB, BDM, BP-12	Lotion, cream, lipstick	HPLC-MS/MS (ESI); type of column: C_18_; column temp.: 30 °C; mobile phase: gradient methanol/0.1% ammonium hydroxide (aq)	LOD: 2–20 mg kg^−1^ LOQ: 5–50 mg kg^−1^ R: 86.9–103.5% RSD: 1–6.8%	[29]
EHS, EMC, BP-3, OC, EMT, BDM, DHHB, ET, DBT	Cream	UHPSFC-PDA; type of column: Torus 2-PIC; column temp.: 40 °C; mobile phase: gradient CO_2_/methanol/water/ammonium acetate	LOD: 0.2–1.7 mg kg^−1^ LOQ: 1–10.8 mg kg^−1^ R: 97.5–103.2% RSD: 0.7–1.6%	[30]
BP-1, BP-2, BP-3, BP-8, HBP	Toothpaste, shampoo, face cleansers, sunscreens, body lotions, gels, hair gels,lotions, mask, hand sanitizer	HPLC-MS/MS (ESI^-^); type of column: C_18_; column temp.: 40 °C; mobile phase: gradient methanol/acetonitrile/water	LOD: 0.002–0.197 ng mL^−1^ LOQ: 0.001–0.059 ng mL^−1^ R: 61.9–116% RSD: no data	[31]
BP-1	Nail product	GC-MS/MS (EI^+^); type of column: ZB-SemiVolatiles; oven temp.: 40 °C/2 min—5 °C/1 min to 65 °C—50 °C/1 min to 300 °C/5 min	LOD: 18.3–2370 µg g^−1^ LOQ: no data R: 101–105% RSD: 0.69–1.13%	[32]
BDM, EMT, OMC, OC, ET	Lotion	HPLC-UV/Vis; type of column: C_18_; mobile phase: acetonitrile/0.25% formic acid (aq)	LOD: 15 ng mL^−1^ LOQ: no data R: 88.1–104.7% RSD: 0.8–5.4%	[33]
BDM	Emulsion	LC-UV/Vis; type of column: C_18_; column temp.: 42 °C; mobile phase: acetonitrile/0.5% phosphoric acid (aq)	LOD: 0.05796 µg mL^−1^ LOQ: 0.19322 µg mL^−1^ R: no data RSD: 0.46–2.83%	[34]
EMC, MBC, BP-1, BP-2, BP-6, BP-4, OC, PABA, EDP, EHS, HS, IMC, BP-3, BP-8, BS, MA	Cream, nail polish, lipstick, hair gel	GC-MS/MS (EI^+^); type of column: SLB-5 ms; oven temp.: 100 °C/1 min—25 °C/1 min—290 °C/5 min	LOD: 0.0027–0.56 µg g^−1^ LOQ: 0.009–1.9 µg g^−1^ R: 37.4–110.5% RSD: 3.9–9.1%	[35]
ET	Cream, lotion	TLC-DS.; type of layer: silica gel; mobile phase: cyclohexanediethyl ether (1:1)	LOD: 0.03 μg spot^−1^ LOQ: 0.1 μg spot^−1^ R: 95–105% RSD: 4.5–5%	[36]
PMDSA, BDM, OC, EHS	Cream	HTLC; type of column: C_18_; column temp.: 150–200 °C; mobile phase: isocratic methanol/water	LOD: no data LOQ: no data R: 90.3–113.2% RSD: 2.8–5%	[37]
EMC, MBC, BP-1, BP-2, BP-6, BDM, BP-4, PMDSA, MA, OC, EDP, IMC, BP-3, BP-8,	Lipsticks, hair gel, cream, nail polish	HPLC-MS/MS; type of column: C_18_; oven temp.: 30 °C; mobile phase: gradient methanol/0.1% formic acid/ammonia (aq)	LOD: 0.00039–0.031 µg g^−1^ LOQ: 0.0013–0.1 µg g^−1^ R: 81.7–102% RSD: 4.5–13%	[38]
BDM, BP-3, EMC, EMT	Emulsion	HPLC-UV/Vis; type of column: C_18_; column temp.: 25 °C; mobile phase: gradient tetrahydfofuran/acetonitrile/acetic acid (aq)	LOD: no data LOQ: no data R: 99.2–104.8% RSD: no data	[40]
BP-4	Shampoo	TLC-UV/Vis; type of layer: silica gel 60 plates; mobile phase: acetate/ethanol/water/phosphate buffer (15:7:5:1; *v*/*v*/*v*/*v*)	LOD: 0.03 μg spot^−1^ LOQ: 0.1 μg spot^−1^ R: 100–103% RSD: 0.58–1.99%	[41]
EHS, EMC, BP-3, OC, BDM, DHHB, ET, DBT	Cream	SFC-UV/Vis; type of column: 2-ethyl pyridine; column temp.: 30 °C; mobile phase: gradient CO_2_/methanol/ethanol (97:1.5:1.5)	LOD: no data LOQ: no data R: no data RSD: 0.6–2%	[42]

^a^ LOD and LOQ expressed as: *w*/*w* when referred to sample or *w*/*v* when referred to sample solution.

**Table 3:** Published papers on UV filters determination in urine.

UV Filters	Extraction Technique	Analytical Technique	Analytical Performance	Comments	Ref.
BP-3	SPE (C_8_)	HPLC-UV/Vis; type of column: C_18_; mobile phase: isocratic methanol/water (70:30)	No data	Total content	[44]
BP-3	SPE (Bond Elut Certify LRC)	UPLC-MS/MS (ESI^-^); type of column: Kinetex Phenyl-Hexyl; column temp.: 35 °C; mobile phase: water/acetonitrile/acetic acid (aq)	LOD: 0.3 ng mL^−1^ LOQ: 0.61–200 ng mL^−1^ R: 75.8–80.3% RSD: 0.3–8%	Total and free forms content	[46]
BP-3	Online SPE (RP_18_)	HPLC-MS/MS (APCI^−^); type of column: RP_18_; mobile phase: gradient methanol/water	LOD: 0.3–0.5 ng mL^−1^ LOQ: no data R: 97–105% RSD: 1.7–20%	Total and forms content	[47,48,49]
BP-3	SPE (C_18_)	HPLC-MS (APCI); type of column: C18-PFP; mobile phase: methanol/water	LOD: 0.2 ng mL^−1^ LOQ: no data R: 96% RSD: 9.03–11.7%	Total content	[50]
BP-1, BP-2, BP-8, 4-OH-BP	LLE (solvent: ethyl acetate)	HPLC-MS/MS (ESI^+^/ ESI^−^); type of column: C_18_; mobile phase: methanol/water (90:10; *v*/*v*)	LOD: no data LOQ: 0.7–2.0 ng mL^−1^ R: 84–112% RSD: no data	Total content	[51]
BP-3	SDME (acceptor phase:[C6MIM][PF6]; 25 min; 900 rpm)	LC-UV; type of column: RP_18_; mobile phase: ethanol/1% acetic acid aq (60:40; *v*/*v*)	LOD: 1.3 ng mL^−1^ LOQ: no data R: no data RSD: 6%	Free forms	[52]
BP, BP-OH, 2-OH-BP, BP-3, BP-10	SBSE (PDMS; 60 min; 500 rpm)	GC-MS; type of column: DB-5 ms; oven temp.: 40 °C/1 min—5 °C/1 min to 190 °C—15 °C/1 min to 280 °C/3 min	LOD: 0.05–0.1 ng mL^−1^ LOQ: 0.2–0.5 ng mL^−1^ R: 98.7–101.7% RSD: 1.5–4.8%	Free forms	[53]
BP, BP-OH, 2-OH-BP, BP-3, BP-10	HFLPME (toluene; 15 min; 500 rpm)	GC-MS (EI); type of column: DB-5 ms; oven temp.: 40 °C/ 1 min—5 °C/ 1 min to 190 °C—15 °C/1 min to 280 °C/ 4 min	LOD: 5–10 pg mL^−1^ LOQ: 20–50 pg mL^−1^ R: 89.3–100.2% RSD: 2.5–9.3%	Total content	[54]
BP-1, BP-3, BP-8, BP-2, 4-OH-BP	LLE (solvent; 50% MTBE/ethyl acetate)	HPLC-MS/MS (ESI^−^); type of column: C_18_; mobile phase: gradient methanol/water	LOD: 0.08–0.28 mg mL^−1^ LOQ: 0.28–0.9 mg mL^−1^ R: 85.2–99.6% RSD: 2.8–4.5%	Total content	[55]
BP-1, BP-3, BP-8, THB	SPE (C_18_)	LC-MS/MS (ESI^+^); type of column: Mediterranean SEA 18; mobile phase: gradient methanol/water/0.1% formic acid aq	LOD: 1 ng mL^−1^ LOQ: 2–4 ng mL^−1^ R: 84–111% RSD: no data	Total content	[56]
BP-1, BP-2, BP-3, BP-8, 4-OH-BP	LLE (solvent; 50% MTBE/ethyl acetate)	HPLC-MS/MS (ESI); type of column: C_18_; mobile phase: gradient methanol/water	LOD: 0.013–0.28 ng mL^−1^ LOQ: no data R: 85.2–99.6% RSD: 1.4–4.5%	Total content	[57]
BP-1, BP-2, BP-3, BP-7, 4-OH-BP, 4-MBP, 4-MBC, 3-BC	LLE	On-line TurboFlow-LC–MS/MS; type of column: TurboFlow Cyclone P and Hypersil Gold aQ	LOD: 0.2–1.0 ng mL^−1^ LOQ: no data R: 77.1–108% RSD: 5.7–15.1%	Total and free form content	[58]
EDP	Automated SPE (C_18_ HD)	LC-MS/MS (ESI^+^); type of column: Mediterranean SEA C_18_; mobile phase: gradient methanol/ acetonitryle/water/0.2% formic acid	LOD: 0.3–1.1 ng mL^−1^ LOQ: 0.9–3.5 ng mL^−1^ R: 91–107% RSD: no data	Total and free forms content	[59]
BP-3, OMC, OS, HS	LLE (solvent: acetonitrile)	HPLC-DAD; type of column: C_18_; mobile phase: gradient methanol/water (75:25; *v*/*v*)	LOD: 0.03–0.2 µg mL^−1^ LOQ: 0.1–0.4 µg mL^−1^ R: 86.8–92.2% RSD: 3.0–4.4%	Total content	[60]
BP-1, BP-2, BP-3, BP-8, 4-OH-BP	DLLME (disperser solvent: acetone; extraction solvent: trichloromethane)	UHPLC-MS/MS	LOD: 0.1–0.2 ng mL^−1^ LOQ: 0.3–0.6 ng mL^−1^ R: 88–104% RSD: 0.5–22.5%	Total and free forms content	[61]
BP-3, 4-MBC, HS, OC	ASE & SPE	GC-MS/MS	LOD: 0.47–0.59 pg mL^−1^ LOQ: no data R: 70.5–110.7% RSD: <5.04%	Total and free forms content	[62]
BMDBM, CDAA, EHS, 5-OH-EHS, OC	LLE (solvent: actonitrile)	LC-LC-MS/MS (ESI); type of column: RP-18 ADS;	LOD: 0.1–1.5 µg L^−1^ LOQ: 0.2–4.1 µg L^−1^ R: 94.2–113.6% RSD: 2.6–16.5%	Total content	[63]
5OH-EHS, 5oxo-EHS, 5cx-EPS	Online SPE (TurboFlow Phenyl)	HPLC-MS/MS (ESI); type of column: C_18_; mobile phase: gradient acetonitryle/water/0.05% acetic acid	LOD: no data LOQ: 0.01–0.15 µg L^−1^ R: 96–106% RSD: 1.2–2.4%	Total and free forms content	[64]
BP-3	Online SPE (RP_18_)	HPLC-MS/MS (ESI); type of column: XDB-C_18_; mobile phase: gradient methanol/water	LOD: 0.16 µg L^−1^ LOQ: no data R: 101% RSD: 5%	Total and free forms content	[65]
BP-1, BP-2, BP-3, BP-8, 4-OH-BP	LLE (solvent: ethyl *tert*-butyl ether/ethyl acetate (5:1; *v*:*v*))	UHPLC-TQMS (ESI^−^); type of column: C_18_; column temp.: 30 °C; mobile phase: water/acetonitrile	LOD: 0.01–0.2 ng mL^−1^ LOQ: no data R: 90.7–110.1% RSD: 6.9–14.2%	Total and free forms content	[66]
BP-1, BP-2, BP-3, BP-8, 4-OH-BP	VADLLME (disperser solvent: 2-propanol; extraction solvent: dichloromethane)	LC-MS/MS; type of column: C_18_; column temp.: 23 °C; mobile phase: water/methanol	LOD: 0.02–0.03 ng mL^−1^ LOQ: 0.05–0.4 ng mL^−1^ R: no data RSD: 1.2–12%	Total content	[67]
BP-1, BP-2, BP-3, BP-8, 4-OH-BP	AALLME (extraction solvent: 1,2-dichloroethane)	LC-MS/MS (ESI); type of column: C_18_; column temp.: 40 °C; mobile phase: water/methanol	LOD: 0.02–0.06 ng mL^−1^ LOQ: 0.05–0.20 ng mL^−1^ R: no data RSD: <15%	Total content	[68]
PABA, 4-AHA, 4-AMB, 4-OCH_3_-AHA	LLE & SPE (solvent: ethyl acetate; C_18_)	HPLC-ECD; type of column: C_18_; mobile phase: methanol/phosphate buffer (pH 5.5) (20:80; *v*/*v*)	LOD: no data LOQ: 0.04–0.18 ng mL^−1^ R: 96–99% RSD: 0.2–3.8%	Total content	[69]
BP-1, BP-3	SPE (C_8_)	HPLC-UV; type of column: C_18_; mobile phase: acetonitryle/water	LOD: 2–40 ng mL^−1^ LOQ: no data R: no data RSD: 6.6–13%	Total and free form content	[70]
PMDSA	Online SPE	SIA-FL	LOD: 12 ng mL^−1^ LOQ: no data R: no data RSD: 2–13%	Free forms	[71]
PEG-25 PABA	SPE (C_18_)	LC-FL; mobile phase: dimethylfuran	LOD: 2.6 ng mL^−1^ LOQ: no data R: 91–100% RSD: 3–10%	Total content	[72]
BP-4, 4-DHB, BP-2, BP-1, BP-8, BZ	FPSE	HPLC-PDA; type of column: C_18_; mobile phase: methanol/phosphate buffer (pH 3) (45:55; *v*/*v*)	LOD: 0.03 µg mL^−1^ LOQ: 0.1 µg mL^−1^ R: no data RSD: 2.3–14.4%	Total content	[73]
EDP	In-vial MMLLE (hydrophobic PTFE membranes)	GC-MS; type of column: SPB-5; oven temp.: 60 °C/1.5 min—30 °C/1 min to 275 °C/20 min	LOD: no data LOQ: 0.11 µg L^−1^ R: no data RSD: 7.4%	Total content	[74]
BP-3, BP-4	SI SPE (C_18_ and diethylaminopropyl)	LC/UV; type of column: RP_18_; mobile phase: ethanol/acetate buffer/1% acetic acid	LOD: 30–60 ng mL^−1^ LOQ: no data R: no data RSD: 6–13%	Free forms	[75]
BP-1, BP-2, BP-8, 4-OH-BP	MEPS (C_18_)	LC-MS/MS; mobile phase: water/methanol	LOD: 0.005–0.03 ng mL^−1^ LOQ: 0.02–0.10 ng mL^−1^ R: 18–118% RSD: 1–16%	Total and free forms content	[78]
BP-1, BP-3, BP-8	SPME (Carbowax/DVB)	GC-MS; type of column: DB5-MS; Oven temp.: 50 °C/0.1 min—30 °C/1 min to 150 °C—18 °C/1 min to 250 °C/12 min	LOD: 5–10 ng mL^−1^ LOQ: no data R: no data RSD: 5–8%	Total content	[79]
BP, BP-1, BP-3, 4-OH-BP	BAµE	HPLC–DAD; type of column: Sea-18; mobile phase: methanol/water (75:25; *v*/*v*)	LOD(P2): <1.0 µg L^−1^ LOQ(P2): <0.3 µg L^−1^ LOD(AC4): <1.3 µg L^−1^ LOQ(AC4): <0.4 µg L^−1^	Total content	[76]
OMC, BP-3, OC, OS, HS	DLLME (disperser solvent: carbon tetrachloride; extraction solvent: acetonitrile)	HPLC-DAD; type of column: C_18_; mobile phase: isocratic water/methanol/acetonitrile (8:42:50; *v*/*v*/*v*)	LOD: no data LOQ: 3–45 ng mL^−1^ R: 86.9–97.3% RSD: 0.1–6.4%	Total content	[77]
BP-1, BP-2, BP-3, BP-8, 4-OH-BP	Microextraction using a monolithic stirring extraction unit (150 min; 1100 rpm)	UPLC-DAD; mobile phase: acetonitrile/water	LOD: 1–10 µg L^−1^ LOQ: 5–20 µg L^−1^ R: 71–114 % RSD: 5.6–9.1%	Total content	[80]

**Table 4:** Published studies on UV filters determination in blood, plasma, and serum.

UV Filters	Matrix	Extraction Technique	Analytical Technique	Analytical Performance	Comments	Ref.
BP-3, BP-1, BP-8	Serum	DLLME (disperser solvent: acetone: extraction solvent: chloroform)	LC-MS/MS (ESI^+^); type of column: C_18_; mobile phase: gradient methanol/water/0.1% formic acid	LOD: 7–8 µg L^−1^ LOQ: 22–28 µg L^−1^ R: 77–104% RSD: 8–9%	Total content	[45]
BP-3, OMC, OS, HS	Plasma	LLE (solvent: acetonitrile)	HPLC-DAD; type of column: C_18_; mobile phase: gradient methanol/water (75:25; *v*/*v*)	LOD: 0.03–0.2 µg mL^−1^ LOQ: 0.1–0.4 µg mL^−1^ R: 90.8–103.8% RSD: 2.1–4.4%	Total content	[60]
BP-3, OMC, OS, HS	Bovine serum albumin	LLE (solvent; acetonitrile)	HPLC-DAD; type of column: C_18_; mobile phase: gradient methanol/ water (75:25; *v*/*v*)	LOD: 0.03–0.2 µg mL^−1^ LOQ: 0.1–0.4 µg mL^−1^ R: 97.9–102.3% RSD: 1.2–3.3%	Total content	[60]
BP-1, BP-2, BP-3, BP-6, BP-8, 4-OH-BP	Menstrual blood	DLLME (disperser solvent: acetone; extraction solvent: trichloromethane)	UHPLC-MS/MS (ESI); type of column: C_18_;	LOD: 0.2–0.3 ng mL^−1^ LOQ: no data R: no data RSD: 0.28–1.59%	Total and free forms content	[82]
BP-1, BP-2, BP-3, BP-6, BP-8, 4-OH-BP	Serum	DLLME (disperser solvent: acetone; extraction solvent: trichloromethane)	UPLC-MS/MS (ESI^+^); type of column: C_18_; mobile phase: gradient 0.1% ammoniacal aq/0.1% ammonia in methanol	LOD: 0.1–0.3 ng mL^−1^ LOQ: 0.4–0.9 ng mL^−1^ R: 97–106% RSD: 1.9–13.7%	Total and free forms content	[83]
BP-3	Serum	Online SPE	HPLC-MS/MS (APPI^-^)	LOD: 0.5 ng mL^−1^ LOQ: no data R: 96% RSD: 7.7–8.7%	Total content	[84,85]
OC, BMDBM, CDAA	Plasma	LLE (solvent: acetonitrile)	LC-LC-MS/MS (ESI); type of column: C_18_; mobile phase: methanol/water	LOD: 1.1–6.5 µg L^−1^ LOQ: 3.5–20.7 µg L^−1^ R: 89.0–112.8% RSD: 3.0–4.9%	Total content	[63]
BP-3	Plasma	LLE (solvent: acetonitrile)	UHPLC-DAD; type of column: C_18_; mobile phase: acetonitrile/water	LOD: no data LOQ: no data R: 94–99% RSD: 2.3–4.6%	Total content	[86]
BP-4, 4-DHB, BP-2, BP-1, BP-8, BZ	Whole blood	FPSE	HPLC-PDA; type of column: C_18_; mobile phase: methanol/phosphate buffer (pH 3) (45:55; *v*/*v*)	LOD: 0.03 µg mL^−1^ LOQ: 0.1 µg mL^−1^ R: no data RSD: 0.4–10.8%	Total content	[73]
BP-4, 4-DHB, BP-2, BP-1, BP-8, BZ	Plasma	FPSE	HPLC-PDA; type of column: C_18_; mobile phase: methanol/phosphate buffer (pH 3) (45:55; *v*/*v*)	LOD: 0.03 µg mL^−1^ LOQ: 0.1 µg mL^−1^ R: no data RSD: 3.6–11.1%	Total content	[73]
BP-3, BP-1, 4-OH-BP, BP-8, 4-DHB, BP-2, BP-4, BMDBM	Umbilical cord blood	LLE (solvent: MTBE)	LC-MS/MS (ESI^+^; ESI^−^); type of column: R_18_; mobile phase: methanol/water	LOD: 0.05–0.42 ng mL^−1^ LOQ: 0.18–1.39 ng mL^−1^ R: 14.3–146.4% RSD: 0.5–33.8%	Total content	[81]
BP, 4-MBP	Plasma	LLE-SPE (solvent: MTBE; Oasis Prime-HLB)	HPLC-MS/MS (ESI); type of column: C_18_; mobile phase: 0.1% formic acid in water/0.1% formic acid in methanol	LOD: 0.8–2 pg mL^1^ LOQ: 3.5–7 pg mL^−1^ R: 87–97% RSD: 3.1–9.1%	Total content	[87]

**Table 5:** Published studies on UV filters determination in semen, saliva, milk, nail, and placental tissue.

UV Filters	Matrix	Extraction Technique	Analytical Technique	Analytical Performance	Comments	Ref.
BP-1, BP-3, BP-8, THB	Semen	SPE (C_18_)	LC-MS/MS (ESI^+^); type of column: Mediterranean SEA 18; mobile phase: gradient mobile phase: 0.1% formic acid in water/0.1% formic acid in methanol	LOD: 0.03–0.04 ng mL^−1^ LOQ: 0.08–0.13 ng mL^−1^ R: 98–115% RSD: no data	Total content	[56]
BP-3, OMC, OS, HS	Epidermal membranes	LLE (solvent: acetonitrile)	HPLC-DAD; type of column: C_18_; mobile phase: gradient methanol/water (75:25; *v*/*v*)	LOD: 0.03–0.2 µg mL^−1^ LOQ: 0.1–0.4 µg mL^−1^ R: 98.5–99.5% RSD: 1.8–3.2%	Total content	[60]
OC, 3-BC, 4MBC, OMC, EDP, BP-1, BP-3, BP-6, BP-8, 4-OH-BP	Milk	QuEChERS Extraction; SALLE & d-SPE (sorbent: polysecondary amine and magnesium sulphate)	UHPLC-MS/MS (API); type of column: C_18_; mobile phase: gradient acetonirile/water/0.1% formic acid	LOD: 0.1–0.2 ng mL^1^ LOQ: 0.4–0.6 ng mL^−1^ R: 87–112% RSD: 8–14%	Total content	[88]
BP-3	Breast milk	Online SPE (RP_18_)	HPLC-MS/MS (APCI^-^); type of column: RP_18_; mobile phase: gradient methanol/water	LOD: 0.51 ng mL^−1^ LOQ: no data R: 94.7% RSD: 12.7–18%	Total and free forms content	[89]
BP-1, BP-3, 4-OH-BP, 4DHB, 4MBC, ODPABA, EtPABA, TBHPBT	Breast milk	Online TFC	HPLC-MS/MS (ESI); type of column: Cyclone and C_18_; mobile phase: gradient methanol/water/0.1% formic acid	LOD: 0.1–1.5 ng g^−1^ LOQ: 0.3–5.1 ng g^−1^ R: no data RSD: 1–12%	Total content	[90]
BP-3	Milk	Online SPE (RP_18_)	HPLC-MS/MS (APCI^−^); type of column: RP_18_; mobile phase: methanol/water	LOD: 0.4 ng mL^−1^ LOQ: no data R: 102% RSD: 8.8–12%	Total and free forms content	[91]
BP-1, BP-3, BP-6, BP-8, 4-OH-BP	Breast milk	USAD-SPE (15 min of sonification; sorbents: C_18_, polysecondary amine and magnesium sulphate)	UHPLC-MS/MS (ESI^+^); type of column: C_18_; mobile phase: gradient aqueous ammonium formate solution (pH 9)/0.025% ammonia in MeOH	LOD: 0.1–0.2 ng mL^−1^ LOQ: 0.3–0.6 ng mL^−1^ R: 90.9–109.5% RSD: 2.0–12.3%	Total content	[92]
BP-1, BP-2, BP-3, BP-6, BP-8, 4-OH-BP, THB, AVB	Nail	MAE (20 min, 1000 W of power)	UHPLC-MS/MS (ESI^+^); type of column: C_18_; mobile phase: gradient methanol/water/0.1% formic acid	LOD: 0.2–1.5 ng g^−1^ LOQ: 1.0–5.0 ng g^−1^ R: 90.2–112.2% RSD: 0.8–12.3%	Total content	[93]
BP-1, BP-2, BP-3, BP-6, BP-8, 4-OH-BP	Placental tissue	MSPD (solvent: ethyl acetate)	UHPLC-MS/MS (ESI); type of column: C_18_; mobile phase: gradient 0.1% ammoniacal aq solution/0.1% ammonia in methanol	LOD: 0.1 ng g^−1^ LOQ: 0.2–0.4 ng g^−1^ R: 95–106% RSD: 4.5–11.8%	Free forms	[94]
BP-1, BP-2, BP-3, BP-4, 4-OH-BP	Placental tissue	SLE (solvent: ethyl acetate)	LC-MS/MS (ESI^−^); type of column: RP_18_; mobile phase: gradient methanol/water	LOD: 0.02–0.36 ng mL^−1^ LOQ: 0.05–1.20 ng mL^−1^ R: 72–110% RSD: 4–40%	Total content	[95]
BP-1, BP-2, BP-3, BP-8, 4-OH-BP	Saliva	DLLME (disperser solvent: acetone; extraction solvent: trichloromethane)	LC-MS/MS; type of column: C_18_; mobile phase: gradient methanol/water	LOD: 0.01–0.15 ng mL^−1^ LOQ: 0.05–0.40 ng mL^−1^ R: no data RSD: 1–19%	Total content	[96]
EDP, 3-BC, MBC, OMC, OC, BP-1, BP-3, BP-6, BP-8, 4-OH-BP	Placenta tissue	UAE (disperser solvent: methanol; extraction solvent: anisole; 3 min of sonification)	UHPLC-MS/MS; type of column: C_18_; mobile phase: gradient acetonitrile/0.25% formic acid aq	LOD: 0.05–0.2 µg kg^−1^ LOQ: 0.15–0.5 µg kg^−1^ R: 90–112% RSD: 3–15%	Total content	[97]

## Data Availability

Not applicable.

## Abbreviations

[C_6_MIM][PF_6_]:	hexyl-3-methylimidazolium hexafluorophosphate
2-OH-BP:	2-hydroxybenzophenone
3-BC:	3-benzophenone camphor
4-AHA:	p-aminohippuric acid
4-AMB:	p-acetamidobenzoic acid
4-DHB:	4,4-dihydroxybenzophenone
4-MBC:	3-(4-methylbenzylidene)-camphor
4-OCH_3_-AHA:	p-acetamidohippuric acid
4-OH-BP:	4-hydroxybenzophenone
5cx-EPS:	5-(((2-hydroxybenzoyl)oxy)methyl)heptanoic acid
5-OH-EHS:	5-hydroxy-2-ethylhexyl salicylate
5oxo-EHS:	2-ethyl-5-oxohexyl 2-hydroxybenzoate
AALME:	air-assisted liquid–liquid microextraction
Ac:	Acetone
APCI:	atmosphere pressure chemical ionisation
API:	atmosphere pressure ionisation
APPI:	atmosphere pressure photoionisation
ASE:	accelerated solvent extraction
BMDBM:	butyl methoxydibenzoylmethane/avobenzene
BAµE:	bar adsorptive microextraction
BC:	benzyl cinnamate
BDM:	butyl methoxydibenzoylmethane
EMT:	bis-ethylhexyloxyphenol methoxyphenyl triazine
BP:	Benzophenone
BP-1:	2,4-dihydroxybenzophenone
BP-10:	2-hydroxy-4-methoxy-4′-methylbenzophenone
BP-12:	(2-hydroxy-4-octoxy-phenyl)-phenyl-methanone
BP-2:	2,2′,4,4′-tetrahydroxybenzophenone
BP-3:	2-hydroxy-4-methoxybenzophenone
BP-4:	2-hydroxy-4-methoxybenzophenone-5-sulphonic acid
BP-6:	2,2′-dihydroxy-4,4′-dimethoxybenzophenone
BP-7:	5-chloro-2- hydroxybenzophenone
BP-8:	2,2′-dihydroxy-4-methoxybenzophenone
BP-OH:	Benzhydrol
BS:	benzyl salicate
BZT:	Benzotriazole
C18:	Octadecyl
CDAA:	2-cyano-3,3-diphenyl acrylic acid
CPE:	cloud point extraction
DAD:	diode-array detection
DART-MS:	direct-analysis-in-real-time mass spectrometry
DBT:	diethylhexyl butamino triazone
DCM:	Dichloromethane
DEA:	Diethylaminopropyl
DHHB:	diethyloamino hydroxybenzoyl hexyl benzoate
DLLME:	dispersive liquid–liquid microextraction
DMF:	n,n-dimethylformamide
DTS:	drometrizole trisiloxane
DS:	Densitometry
d-SPE:	dispersive solid-phase extraction
EA:	ethyl acetate
ECD:	electron captur detector
EDP:	2-ethylhexyl 4-(n,n-dimethylamino)benzoate
EHC:	ethylhexyl cinnamate
EHS:	2-ethylhexyl salicylate
EI:	electron impact
EMC:	ethylhexyl methoxycinnamate
EHS:	ethylhexyl salicylate
ESI:	electrospray ionisation
ET:	ethylhexyl triazone
EtOH:	Ethanol
EtPABA:	ethyl p-aminobenzoic acid
FL:	Fluorescence
FPSE:	fabric phase sorptive extraction
GC:	gas chromatography
HFLPME:	hollow-fiber liquid-phase microextraction
HPLC:	high-performance liquid chromatography
HS:	salicylic acid 3,3,5-trimethcyclohexyl ester
HTLC:	high-temperature liquid chromatographic
IMC:	isoamyl p-methoxycinnamate
LC:	liquid chromatography
LD:	liquid desorption
LLE:	liquid–liquid extraction
LOD:	limit of detection
log _Ko/w_:	log octanol/water partition coefficient
LOQ:	limit of quantification
LTP-MS:	low temperature plasma ionisation mass spectrometry
MA:	menthyl anthranilate
MAE:	microwave-assisted extraction
MBBT:	methylene bis-benzotriazolyl tetramethyl butyl phenol
MBC:	4-methylbenzylidene camphor
MBP:	methylene bis-benzotriazoyl tetramethylbutylphenol
MeCN:	Acetonitrile
MEKC:	micellar electrokinetic capillary chromatography
MeOH:	Methanol
MEPS:	microextraction by packed sorbent
MMLLE:	microporous membrane liquid–liquid extraction
MS/MS:	tandem mass spectrometry
MS:	mass spectrometry
MSPD:	matrix solid phase dispersion
MTBE:	methyl tert-butyl ether
NaCl:	sodium chloride
OC:	4-methylbenzilidene camphor/octocrylane
ODP:	octyl dimethyl PABA
ODPABA:	2-ethylhexyl 4-(dimethylamino)benzoate
OMC:	2-ethylhexyl p-methoxycinnamate
OS:	2-ethylhexylsalicylate
PABA:	p-aminobenzoic acid
PMDSA:	2-phenylbenzimidazole-5-sulphonic acid
PDA:	photodiode-array detection
PEG-25 PABA:	polyethylene glycol 25 paminobenzoic acid
PHBA:	4-hydroxy benzoic acid
PLE:	pressurized liquid extraction
p_Ka_	acid dissociation constant
PSA:	primary-secondary amine
QuEChERSExtraction:	Quick, Easy, Cheap, Effective, Rugged, and Safe Extraction
R:	Recovery
RSD:	relative standard deviation
SALLE:	salt-assisted liquid–liquid extraction
SBSE:	stir bar sorptive extraction
SDME:	single-drop microextraction
SFC:	supercritical fluid chromatography
SIA:	sequential injection analysis
SI SPE:	sequential injection solid-phase extraction
SLE:	solid–liquid extraction
SPE:	solid-phase extraction
SPME:	solid-phase microextraction
SWV:	squarewave voltammetry
TBHPBT:	2-(5-tert-butyl-2-hydroxyphenyl)benzotriazole
TCM:	trichloroamine
TFA:	trifluoroacetic acid
TFC:	turbulent flow chromatography
THB:	2,3,4-trihydroxybenzophenone
TLC:	thin-layer chromatography
UAE:	ultrasound-assisted extraction
UHPLC:	ultra-high-performance liquid chromatography
UHPSFC:	ultra-high performance supercritical fluid chromatography
UPLC:	ultra-performance liquid chromatography
USAD-SPE:	ultrasound-assisted dispersive solid phase extraction
UV/Vis:	ultraviolet/visible spectrometry
VADLLME:	vortex-assisted dispersive liquid–liquid microextraction
